# Stem-Cell-Derived Circulating Progenitors Dysfunction in Behçet's Syndrome Patients Correlates With Oxidative Stress

**DOI:** 10.3389/fimmu.2019.02877

**Published:** 2019-12-13

**Authors:** Giacomo Emmi, Amanda Mannucci, Flavia Rita Argento, Elena Silvestri, Augusto Vaglio, Alessandra Bettiol, Alessandra Fanelli, Laura Stefani, Niccolò Taddei, Domenico Prisco, Claudia Fiorillo, Matteo Becatti

**Affiliations:** ^1^Department of Experimental and Clinical Medicine, University of Firenze, Firenze, Italy; ^2^Department of Experimental and Clinical Biomedical Sciences “Mario Serio”, University of Firenze, Firenze, Italy; ^3^Central Laboratory, Azienda Ospedaliero Universitaria Careggi, Firenze, Italy; ^4^Department of Clinical and Experimental Medicine, Center of Sports Medicine, University of Firenze, Firenze, Italy

**Keywords:** Behçet's syndrome, thrombosis, circulating progenitor cells, oxidation, apoptosis

## Abstract

Behçet's syndrome (BS) is a systemic vasculitis considered as the prototype of a systemic inflammation-induced thrombotic condition whose pathogenesis cannot be explained just by coagulation abnormalities. Circulating hematopoietic progenitor cells (CPC), a population of rare, pre-differentiated adult stem cells originating in the bone marrow and capable of both self-renewal and multi-lineage differentiation, are mobilized in response to vascular injury and play a key role in tissue repair. In cardiovascular and thrombotic diseases, low circulating CPC number and reduced CPC function have been observed. Oxidative stress may be one of the relevant culprits that account for the dysfunctional and numerically reduced CPC in these conditions. However, the detailed mechanisms underlying CPC number reduction are unknown. On this background, the present study was designed to evaluate for the first time the possible relationship between CPC dysfunction and oxidative stress in BS patients. In BS patients, we found signs of plasma oxidative stress and significantly lower CD34+/CD45^−/dim^ and CD34+/CD45^−/dim^/CD133+ CPC levels. Importantly, in all the considered CPC subsets, significantly higher ROS levels with respect to controls were observed. Higher levels of caspase-3 activity in all the considered CPC population and a strong reduction in GSH content in CPC subpopulation from BS patients with respect to controls were also observed. Interestingly, in BS patients, ROS significantly correlated with CPC number and CPC caspase-3 activity and CPC GSH content significantly correlated with CPC number, in all CPC subsets. Collectively, these data demonstrate for the first time that CPC from BS patients show signs of oxidative stress and apoptosis and that a reduced CPC number is present in BS patients with respect to controls. Interestingly, we observed an inverse correlation between circulating CPC number and CPC ROS production, suggesting a possible toxic ROS effect on CPC in BS patients. The significant correlations between ROS production/GSH content and caspase-3 activity point out that oxidative stress can represent a determinant in the onset of apoptosis in CPC. These data support the hypothesis that oxidative-stress-mediated CPC dysfunctioning may counteract their vascular repair actions, thereby contributing to the pathogenesis and the progression of vascular disease in BS.

## Introduction

Behçet's syndrome (BS) is a systemic vasculitis of unknown etiology characterized by muco-cutaneous and ocular manifestations as well as articular, neurological, and gastrointestinal involvements (1). Vascular involvement is also present, and represents one of the more important manifestations in terms of morbidity and mortality (2). BS affects both veins and arteries of all sizes and is usually more frequent and severe in young males (3). To date, BS may be considered as the prototype of a systemic inflammation-induced thrombotic condition. Although some studies showed different hemostatic system defects in BS (3, 4), current data indicate that the pathogenesis of thrombosis in BS cannot be explained by coagulation abnormalities only (5). Indeed, neutrophils are pivotal in promoting thrombo-inflammation by producing high amounts of reactive oxygen species (ROS), mainly through NADPH oxidase. This mechanism ultimately leads to a modification of the fibrin clot that becomes less susceptible to plasmin-induced lysis (6). Moreover, in BS patients, endothelial injury plays a prominent role in the onset of thrombosis and inflammation leads to thrombosis also *via* endothelial damage and endothelial cell dysfunction (7). Altogether, these mechanisms may partly explain why immunosuppressive treatment is essential in the management of thrombosis occurring in BS, while anticoagulation generally displays limited effects (8).

Circulating hematopoietic progenitor cells (CPC) are a population of rare, pre-differentiated adult stem cells that originate in the bone marrow and are uniquely capable of both self-renewal and multi-lineage differentiation, including cardiomyocytes, smooth muscle cells, endothelial progenitor cells (EPC) and endothelial cells. CPC possess the ability to be mobilized in response to vascular injury and play a key role in tissue repair (9, 10). CPC replenish specialized somatic cells and maintain the normal turnover of regenerative tissues and organs, such as blood and skin. Interestingly, low circulating CPC number and reduced CPC function are associated with cardiovascular disease and mortality (11, 12).

Circulating CPC are involved in the regulation and repair of the endothelium and in vessel formation (13, 14). Indeed, enhanced mobilization of CPC into the blood has been associated with increased endothelial function and repair (11). However, circulating CPC number and function are dramatically altered when cardiovascular risk factors are present (14, 15). On the other hand, while acute inflammation increases CPC, a chronic inflammatory state might be accompanied by a progressive CPC reduction (16, 17). It has been demonstrated that oxidative stress represents one of the main determinant of CPC number reduction and dysfunction in cardiovascular diseases (18, 19). Upon ROS production inhibition, the observed CPC alterations have been reverted (18, 19). However, the underlying mechanisms of CPC reduction have not been well-understood.

To date, insufficient, and conflicting clinical data to document the CPC number/function in BS patients exist (20, 21). Therefore, the present study was designed to evaluate for the first time the possible relationship between CPC dysfunction and oxidative stress in BS patients.

## Materials and Methods

### Study Population

This was a case–control study. Sixty-one consecutive patients with BS who attended the Behçet Center of the University Hospital of Firenze, Italy, were matched 1:1 for age and sex with healthy control subjects. Patients with other autoimmune diseases and active infectious or neoplastic conditions were excluded, as well as pregnant patients. Control subjects were excluded if they had a history of cerebro- and/or cardiovascular diseases, peripheral arteriopathy, venous thrombo-embolism events, or cancer. Both patients and control subjects were assessed for the presence of vascular risk factors and drug use.

The study protocol was approved by the local Ethical Committee and informed consent was obtained from all subject enrolled.

### Blood Collection

Blood samples were obtained from an antecubital vein in the morning after an overnight fasting and were collected into evacuated plastic tubes (BD Vacutainer Systems, Plymouth, UK) containing ethylenediaminetetraacetate 0.17 mol/L for CPC evaluation.

Because inflammatory events are known to influence CPC number (16), blood was withdrawn after excluding the occurrence of infectious events, defined according to previously published criteria (22), in the previous 15 days.

### Flow Cytometric Analysis of CPC Oxidative Stress and Apoptosis

CPC number was assessed by flow cytometry as previously described with minor modifications (16, 22, 23). Briefly, 200 μl of peripheral venous blood was incubated for 20 min in the dark with the appropriated monoclonal antibodies (PE anti-human CD34, BD Pharmigen, Becton Dickinson, San Jose, CA; APC anti-human CD133, Miltenyi Biotec GmbH, Bergisch Gladbach, Germany; APC-Cy7 anti-human CD45 BD, Becton Dickinson, San Jose, CA). Then, 4 ml of BD FACS Lysing Solution (Becton Dickinson Biosciences, San Jose, CA, USA) was added, gently mixed, and incubated at RT in the dark for 10 min, following the manufacturer's protocol. Then, the cells were centrifuged, the supernatant was discarded, and cells were washed twice in PBS. To determine the level of intracellular ROS generation, cells were incubated with H_2_DCFDA (10 μM) (Invitrogen, CA, USA) in RPMI without serum and phenol red for 15 min at 37°C. After labeling, cells were washed and resuspended in PBS and immediately analyzed by FACS.

To determine the level of Caspase-3 activity, single-cell suspensions were incubated in RPMI without serum and phenol red with FAM-FLICA™ Caspase-3 solution (CaspaseFLICA kit FAM-DEVD-FMK) for 30 min at 37°C, following the manufacturer's protocol, and then washed twice with PBS and immediately analyzed by FACS.

To determine the level of intracellular GSH content, single-cell suspensions were incubated in RPMI without serum and phenol red with 5-chloromethylfluoresceindiacetate, CMFDA (10 μM), for 30 min at 37°C, washed twice with PBS, and analyzed immediately by FACS.

A total of 300,000 cells within the leukocyte gate were acquired using a FACSCanto analyzer (Becton Dickinson, San Jose, CA). Data were processed using BD FacsDiva software. By using a modification of the International Society of Hematotherapy and Graft Engineering guidelines (24), CPC were defined as cells forming a cluster with low side scatter, low-to-intermediate CD45 staining, positive for CD34, CD133, and CD34/CD133.

### Protein Concentration Assay

Protein concentration in the samples was determined using the Bradford assay (25). A standard curve of bovine serum albumin (0–15 μg protein/200 μl volume) was used.

### Protein Carbonyl Content (PC)

Oxidative modification on plasma proteins was assessed on the basis of carbonyl content using 2-4 dinitrophenylhydrazine, as described by Levine et al. (26).

Samples were diluted to obtain a protein concentration of 10 mg/ml, and 100 μl of each sample was aliquoted in Eppendorf tubes. For each sample, a blank measurement was prepared. Then, 400 μl of a DNPH solution (5 mM in 2.5 M HCl) was added to tubes. Blank tubes were also prepared, adding the HCl solution without DNPH. Then, all the tubes were incubated in the dark for an hour, vortexing every 15–20 min. After incubation, protein content was precipitated by adding 500 μl of a 20% trichloroacetic acid (TCA) solution, placing tubes on ice for 5 min, and centrifuging at 10,000 *g* for 5 min to pellet protein content. The supernatant was discarded and the pellet was washed once with 500 μl of 10%TCA, and then twice with 500 μl of a 1:1 solution of ethanol-ethyl acetate. Finally, the pellet was resuspended in guanidine hydrochloride at 37°C for 15 min and the absorbance of carbonyl-bound DNPH was read at 370 nm. The corrected absorbance was calculated subtracting the mean of blank values from raw DNPH values. Then, the concentration was determined using an extinction coefficient of 0.022 μM^−1^ cm^−1^, and normalized with the total protein content.

### TBARS (Thiobarbituric Acid Reactive Substances) Estimation

Plasma TBARS levels were measured using a TBARS assay kit (OXI-TEK, ENZO, USA) as previously reported (27). Briefly, the adduct generated by reacting malondialdehyde with thiobarbituric acid after 1 h at 95°C was measured spectrofluorimetrically, with excitation at 530 nm and emission at 550 nm. TBARS were expressed in terms of malondialdehyde equivalent (nmol/ml) and then normalized for protein concentration.

### Total Antioxidant Capacity (TAC) Assay

The ORAC method (oxygen radical absorbance capacity) was performed as previously described on plasma samples (28). Briefly, fluorescein solution (6 nM) was prepared daily in 75 mM sodium phosphate buffer (pH 7.4) and Trolox (250 μM final concentration) was used as a standard. Seventy microliters of each sample with 100 μl of fluorescein was pre-incubated for 30 min at 37°C in each well, before rapidly adding AAPH solution (19 mM final concentration). Fluorescence was measured using Synergy H1 microplate reader (BioTek, Winooski, VT). Results were expressed as Trolox Equivalents (μM) and then normalized for protein concentration.

### Statistical Analysis

To assess the statistical significance of differences in clinical data and progenitor cell numbers between patients with BS and control subjects, the χ^2^ test for categorical variables and Mann–Whitney test for numeric variables were used. Logistic regression analysis, including age, drug use, and sex as variables possibly influencing the cell number, was performed to test the independency of associations. In this analysis, the logarithm of the cell number was used for a better evaluation of the OR. All analyses were performed using the SPSS (Statistical Package for Social Sciences, Chicago, IL) software for Windows (Version 15.0).

## Results

All the patients enrolled in the study fulfilled the International Criteria for Behçet Disease (ICBD) (29). At the beginning of the disease, almost all the patients presented oral ulcers (96.7%), followed by cutaneous and articular involvement (65.6 and 59%, respectively). More than one third of the patients also had ocular and intestinal manifestations, as well as genital ulcers and vascular involvement. HLA-B51 was present in 42.6% of the patients.

All the patients with a Behçet Disease Activity Form (BDCAF) with a score ≥ 1 were considered active, while BS patients with a BDCAF equal to 0 were defined inactive.

Only a minority of the patients had no treatment at the time of the enrollment or were on corticosteroid as the unique therapy (11.5 and 4.9%, respectively). The majority of the BS patients were on Disease Modifying Anti Rheumatic Drugs (DMARDs) (32.8%) or on biologic +/– traditional DMARDs (50.8%).

Demographic and clinical features of the population studied are summarized in detail in Table 1.

**Table 1:** Main clinical and demographic features of the patients enrolled in the study.

	***N* (% out of 61)**
***N*** **obs**	61
**Sex**	
Male	32 (52.5)
Female	29 (47.5)
**Age at diagnosis**	
Median (IQR; range)	35 (26–42)
**HLA-B51**	
Positive	26 (42.6)
**Manifestations at baseline (ICBD criteria)**	
Oral aphthosis	59 (96.7)
Skin involvement	40 (65.6)
Articular involvement	36 (59.0)
Ocular involvement	23 (37.7)
Intestinal involvement	22 (36.1)
Genital aphthosis	21 (34.4)
Vascular involvement	20 (32.8)
Neurologic involvement	17 (27.9)
Positive pathergy test	4 (6.6)
**Disease activity at time of sample collection**	
Not active (BDCAF = 0)	21 (34.4)
Active (BDCAF ≥ 1)	40 (65.6)
**Active manifestations at time of sample collection**	
Oral aphthosis	22 (36.1)
Articular involvement	17 (27.9)
Intestinal involvement	11 (18.0)
Skin involvement	10 (16.4)
Ocular involvement	9 (14.8)
Neurologic involvement	5 (8.2)
Vascular involvement	4 (6.6)
Genital aphthosis	1 (1.6)
**Ongoing pharmacological therapies**	
No treatment	7 (11.5)
Only corticosteroids	3 (4.9)
Traditional DMARDs	20 (32.8)
Biologic (±traditional) DMARDs	31 (50.8)

### Plasma Oxidative Stress

As reported in Table 2, patient plasma displayed significantly higher total PC and TBARS levels compared to healthy controls (*p* < 0.0001 vs. controls).

**Table 2:** Oxidative stress markers.

	**Controls *n* = 61**	**BS patients *n* = 61**	
Plasma PC (nmol/mg)	10.87 ± 3.08	17.75 ± 4.18	*p* < 0.0001
Plasma TBARS (nmol/ml)	0.66 ± 0.11	2.21 ± 0.82	*p* < 0.0001
Plasma TAC (nmol Trolox equivalent/mg of protein)	21.8 ± 3.9	15.2 ± 4.8	*p* < 0.0001

### Levels of Circulating Progenitor Cells

**Figure 1:**
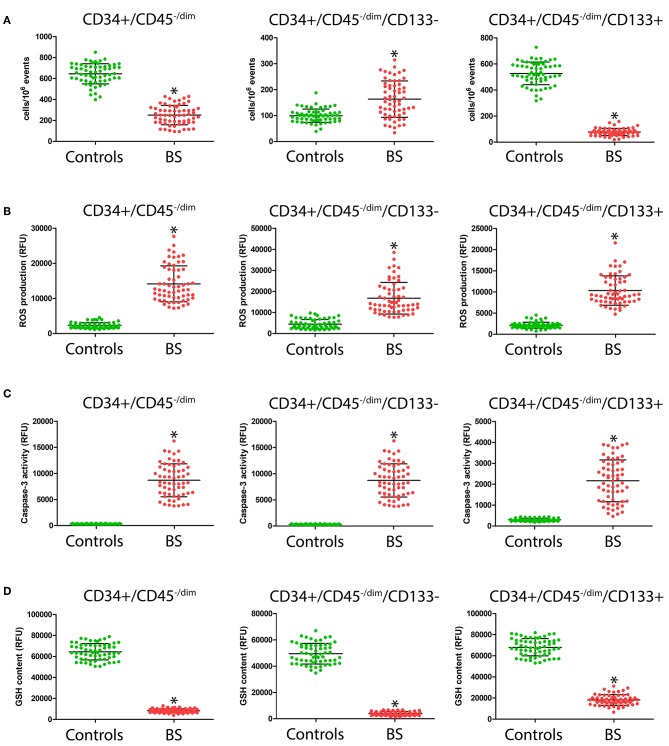
Number **(A)**, intracellular ROS production **(B)**, caspase-3 activity **(C)**, and GSH content **(D)** in CD34+/CD45^−/dim^, CD34+/CD45^−/dim^/CD133– and of CD34+/CD45^−/dim^/CD133+ CPC from patients and controls. ^*^Significant difference vs. control at the *p* < 0.0001 level.

Because several CPC may participate to vascular repair, different phenotypically defined subpopulations of CD34+ CPC were analyzed by FACS analysis, allowing one to determine the level of overall CD34+/CD45^−/dim^ CPC, of CD34+/CD45^−/dim^/CD133– CPC, and of CD34+/CD45^−/dim^/CD133+, representative of more immature CPC. As summarized in Figure 1A, significantly lower CD34+/CD45^−/dim^ and CD34+/CD45^−/dim^/CD133+ CPC levels were observed in BS patients with respect to controls (245 ± 92 vs. 637 ± 96, *p* < 0.0001; 80 ± 28 vs. 536 ± 88, *p* < 0.0001, respectively). On the contrary, CD34+/CD45^−/dim^/CD133– level was significantly higher (*p* < 0.0001) in BS patients with respect to controls (165 ± 70 vs. 101 ± 26).

### CPC Oxidative Stress and Apoptosis

As shown in Figure 1B, in all the considered CPC subsets, we observed significantly higher (*p* < 0.0001) ROS levels in BS patients with respect to controls (CD34+/CD45^−/dim^: 14,333 ± 5104 vs. 2549 ± 794; CD34+/CD45^−/dim^/CD133–: 16,941 ± 7444 vs. 4728 ± 2165; CD34+/CD45^−/dim^/CD133+: 10,396 ± 3469 vs. 2169 ± 737). Likewise, as shown in Figure 1C, we observed significantly higher levels of caspase-3 activity (*p* < 0.0001) in all the considered CPC population in BS patients with respect to controls (CD34+/CD45^−/dim^: 8704 ± 3158 vs. 323 ± 66; CD34+/CD45^−/dim^/CD133–: 12,318 ± 5280 vs. 304 ± 73; CD34+/CD45^−/dim^/CD133+: 2197 ± 1002 vs. 3274 ± 67). A strong reduction in GSH content (Figure 1D) in the CPC subpopulation from BS patients with respect to controls was observed (CD34+/CD45^−/dim^: 8454 ± 1874 vs. 64,792 ± 7825; CD34+/CD45^−/dim^/CD133–: 3993 ± 1407 vs. 48,943 ± 7764; CD34+/CD45^−/dim^/CD133+: 17,598 ± 5101 vs. 67,828 ± 8206).

### Correlation Between Investigated Parameters

**Figure 2:**
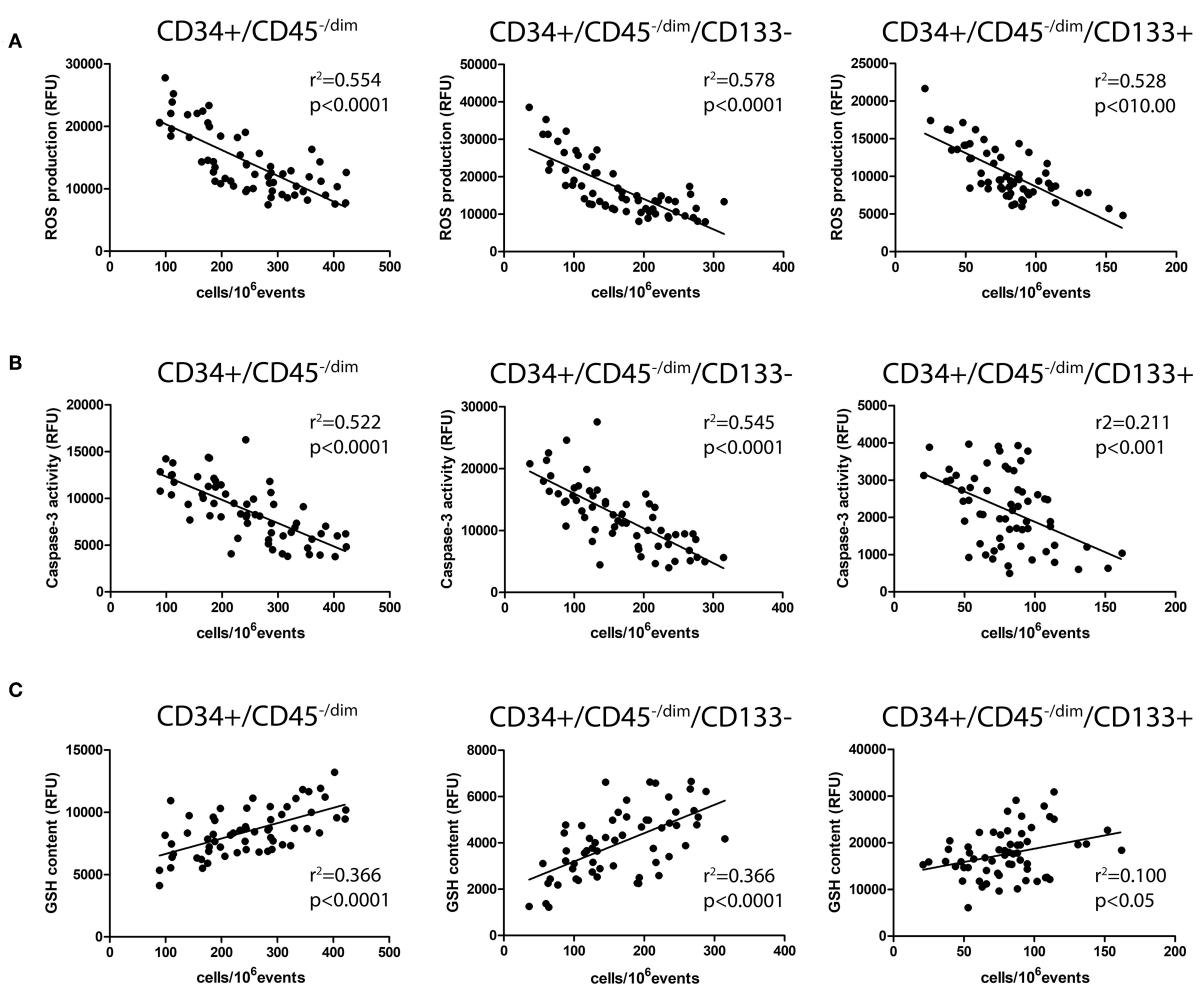
Correlation analysis among CPC number and intracellular ROS production **(A)**, caspase-3 activity **(B)**, and GSH content **(C)**.

As shown in Figure 2A, in all the considered CPC subsets, ROS significantly correlated with CPC number. At the same time, CPC caspase-3 activity (Figure 2B) and CPC GSH content (Figure 2C) significantly correlated with CPC number, in all CPC subsets.

## Discussion

BS is considered the prototype of systemic inflammatory disease causing thrombosis, but the mechanisms underlying the relationship between inflammation and vascular events are far to be elucidated.

In this study, we investigated in a cohort of Behçet's patients the role of CPC, a population of undifferentiated progenitor cells originated in the bone marrow with the ability to be mobilized in response to vascular injury and capable of multi-lineage differentiation including EPC and endothelial cells.

Both EPC and CPC are considered surrogate biomarkers of cardiovascular health since they appear to constitute a natural system for the maintenance of vascular function, improving endothelial repair and neovascularization (30–32). Notably, the restoration of blood supply to ischemic tissues is strictly dependent on endothelial regeneration and angiogenesis. Here, we demonstrate for the first time that CPC from BS patients, but not those from healthy subjects, show signs of oxidative stress and apoptosis. Another important finding emerging from our study is the reduced CPC number observed in BS patients with respect to control subjects. Importantly, the number and function of CPC may reflect the balance between endothelial integrity and repair and can be used as a marker of endothelial function. Indeed, patients with hypertension, coronary artery disease, chronic renal failure, diabetes, sepsis, and rheumatoid arthritis exhibit decreased CPC number (33–36). Moreover, EPC isolated from patients with coronary artery disease and hypertension display an impaired migratory response (34, 35).

The decline in CPC number can be attributed to increased apoptosis, oxidative stress, inflammation, and senescence, in addition to reduced growth and migration from bone marrow (33). However, recent data suggest that increased CPC number may also represent a homoeostatic stress response contributing to vascular damage repair (36, 37). Indeed, in acute coronary syndromes, the early CPC mobilization from the bone marrow seems related to the extension of myocardial ischemia expressed as area at risk (38) and may contribute to the healing process by promoting neovascularization (39).

Moreover, we observed an inverse correlation between circulating CPC number and CPC ROS production, suggesting a possible toxic ROS effect on CPC in BS patients. Indeed, signs of oxidative stress (increased ROS production and reduced GSH content) and apoptosis in CPC from BS patients were observed, suggesting a functional impairment of these cells. Furthermore, the significant correlations between ROS production/GSH content and caspase-3 activity point out that oxidative stress can represent a determinant in the onset of apoptosis in CPC.

To date, few data are available about the possible pathogenetic role of CPC in systemic vasculitis. It was previously reported that the increased number of circulating inflammatory endothelial cells could represent an activity marker in patients with systemic necrotizing vasculitis (40). EPC were reported to be increased in number also in a patient with BS complicated with cerebral trombophlebitis (41). Recently, Bozkirli et al. demonstrated that EPC number was significantly higher in BS patients with thrombosis (42). On the other hand, it was also demonstrated that BS is associated with a progressive reduction in EPC number, which can be interpreted as a mechanism of induction and/or progression of vascular injury in these patients (21). However, to date, there are no data on CPC function in BS patients.

In the case of EPC population, the univocal interpretation of data is limited by the extremely low frequency of the analyzed cell populations and by the lack of validation of the utilized markers. For this reason, in this study, we analyzed the most abundant CPC population instead of the rare EPC population (which accounts for about 0.01 – 0.0001% of nucleated cells). To our knowledge, this is the first study to detect ROS production, GSH content, and caspase-3 activation in CPC, defined as CD34+/CD45^−/low^/CD133+ and CD34+/CD45^−/low^/CD133–, in peripheral blood (not in isolated and cultured cells).

It is accepted that EPC mobilization can be stimulated by transient restricted inflammatory response, while high-grade inflammation results in decreased EPC number and EPC dysfunction (43). Considerable evidence also suggests that ROS play a key role in EPC mobilization/function (44). In particular, low ROS levels activate pro-angiogenic pathways in EPC, whereas high ROS levels impair EPC function. Therefore, oxidative stress is responsible not only for EPC circulating number reduction but also for an impairment EPC function with consequent harmful effects in vascular homeostasis. Indeed, during conditions such as diabetes mellitus, characterized by oxidative stress, the mobilization of dysfunctional EPC is observed (45). Indeed, increased superoxide generation reduces EPC levels and impairs EPC function (46). In addition, incubation of EPC with hydrogen peroxide has been shown to induce apoptosis (47), profoundly reducing EPC number (48). Furthermore, increased ROS production has been associated with reduced EPC levels in a rat model of myocardial infarction (49).

An overall imbalance in blood redox status has been proposed in BS (50). Recently, we demonstrated that neutrophils are responsible for an increased ROS production in BS patients, thus favoring thrombosis through a deep modification of fibrinogen secondary structure (51). Accordingly, in the present study, plasma protein carbonyls and TBARS were markedly and significantly increased in BS patients when compared with control subjects, thus confirming an altered oxidative status in BS patients.

In human vasculature, ROS production is counterbalanced by several antioxidant molecules aimed at ROS scavenging. Intracellular antioxidant enzymes, such as glutathione peroxidase, catalase, and manganese superoxide dismutase, were increased in EPC from healthy subjects with respect to differentiated, mature endothelial cells (52). This is in agreement with our data that show, for the first time, a marked increase (+62%) in ROS production in CD133—population with respect to the more immature CD133+ population, in human peripheral blood. In addition, our results indicate, in CPC from human peripheral blood, a significant reduction in GSH content compared with CPC from control subjects, suggesting that an impairment in antioxidant system can promote CPC sensitivity toward oxidative-stress-mediated apoptosis and consequently reduced CPC number in BS patients. Our observations were supported by the finding that glutathione peroxidase-1-deficient mice exhibited a reduced number and functional activity of progenitor cells (53).

The exact oxidative mechanisms underlying CPC dysfunction has not yet been understood. To date, no study has addressed the question whether redox balancing therapeutic strategies can modify CPC function and number. Only when antioxidant therapies will demonstrate to improve these parameters of CPC biology will a safe conclusion be drawn regarding ROS and CPC relationship in humans.

The results of the present study may have implications in the pathogenesis of thrombotic manifestations in BS. Indeed, CPC have not only been associated with coronary artery disease (54) and atherosclerosis (55). Different from other inflammatory immune-mediated conditions, BS is not associated with accelerated atherosclerosis, despite without having a clear pathogenetic explanation (56). Notably, CPC dysfunction has been also evoked as a potential mechanism in deep vein thrombosis occurrence (57) and aneurysm formation (58), typical clinical features of BS.

Future longitudinal studies on a larger BS population would be helpful in order to explore CPC dysfunction in specific subsets of BS patients. Moreover, functional analysis showing the impact of ROS production on vessel wall of BS patients would be of importance.

However, taking into account that oxidative stress plays an important role in the pathogenesis of all vascular diseases (59), our data support the hypothesis that oxidative-stress-mediated CPC dysfunctioning may counteract their vascular repair actions, thereby contributing to the pathogenesis and the progression of vascular disease in BS patients.

## Data Availability Statement

The datasets generated for this study are available on request to the corresponding author.

## Ethics Statement

The studies involving human participants were reviewed and approved by Comitato Etico Università degli Studi di Firenze, Largo Brambilla 3, Firenze, Italy. The patients/participants provided their written informed consent to participate in this study.

## Author Contributions

GE, AM, FA, ES, AB, CF, and MB were responsible for data collection and analysis. MB, AM, and FA performed experiments. GE, ES, AV, and DP monitored patient inclusion. MB and CF were responsible for protocol development and study funding, and supervised the study. NT, AF, and LS gave critical guidance during the project. MB, GE, and CF designed the experiments and wrote the manuscript. All authors contributed substantially to the critical revision of the manuscript, and gave approval of the final draft.

### Conflict of Interest

The authors declare that the research was conducted in the absence of any commercial or financial relationships that could be construed as a potential conflict of interest.
